# Post-polypectomy surveillance interval and advanced neoplasia detection rates: a multicenter, retrospective cohort study

**DOI:** 10.1055/a-1795-4673

**Published:** 2022-04-11

**Authors:** Amanda J. Cross, Emma C. Robbins, Kevin Pack, Iain Stenson, Matthew D. Rutter, Andrew M. Veitch, Brian P. Saunders, Stephen W. Duffy, Kate Wooldrage

**Affiliations:** 1 Cancer Screening and Prevention Research Group (CSPRG), Department of Surgery and Cancer, Imperial College London, London, United Kingdom; 2 Department of Gastroenterology, University Hospital of North Tees, Stockton-on-Tees, United Kingdom; 3 Faculty of Medical Sciences, Newcastle University, Newcastle-upon-Tyne, United Kingdom; 4 Department of Gastroenterology, New Cross Hospital, Wolverhampton, United Kingdom; 5 Wolfson Unit for Endoscopy, St Mark’s Hospital, London, United Kingdom; 6 Centre for Cancer Prevention, Wolfson Institute of Preventive Medicine, Queen Mary University, London, United Kingdom

## Abstract

**Background** Longer post-polypectomy surveillance intervals are associated with increased colorectal neoplasia detection at surveillance in some studies. We investigated this association to inform optimal surveillance intervals.

**Methods** Patients who underwent colonoscopy and post-polypectomy surveillance at 17 UK hospitals were classified as low/high risk by baseline findings. We compared detection rates of advanced adenomas (≥ 10 mm, tubulovillous/villous, high grade dysplasia), high risk findings (HRFs: ≥ 2 serrated polyps/[adenomas] of which ≥ 1 is ≥ 10 mm or has [high grade] dysplasia; ≥ 5 serrated polyps/adenomas; or ≥ 1 nonpedunculated polyp ≥ 20 mm), or colorectal cancer (CRC) at surveillance colonoscopy by surveillance interval (< 18 months, 2, 3, 4, 5, 6 years). Risk ratios (RRs) were estimated using multivariable regression.

**Results** Of 11 214 patients, 7216 (64 %) were low risk and 3998 (36 %) were high risk. Among low risk patients, advanced adenoma, HRF, and CRC detection rates at first surveillance were 7.8 %, 3.7 %, and 1.1 %, respectively. Advanced adenoma detection increased with increasing surveillance interval, reaching 9.8 % with a 6-year interval ( *P* trend < 0.001). Among high risk patients, advanced adenoma, HRF, and CRC detection rates at first surveillance were 15.3 %, 10.0 %, and 1.5 %, respectively. Advanced adenoma and CRC detection rates ( *P* trends < 0.001) increased with increasing surveillance interval; RRs (95 % confidence intervals) for CRC were 1.54 (0.68–3.48), 4.44 (1.95–10.08), and 5.80 (2.51–13.40) with 3-, 4-, and 5-year intervals, respectively, versus an interval of < 18 months.

**Conclusions** Metachronous neoplasia was uncommon among low risk patients, even with long surveillance intervals, supporting recommendations for no surveillance in these patients. For high risk patients, a 3-year surveillance interval would ensure timely CRC detection.

## Introduction

Colorectal cancer (CRC) can be prevented by removing premalignant polyps (PMPs) 1 . Patients prone to developing metachronous polyps or CRC are recommended to undergo surveillance, usually by colonoscopy, to reduce CRC incidence and mortality 1 2 3 4 . Surveillance should be performed at the minimum frequency to achieve these aims because it is costly, demanding on endoscopy resources, and carries a risk of complications for patients 1 .

National surveillance guidelines classify patients’ risk by baseline polyp characteristics. The UK, US, and European Society of Gastrointestinal Endoscopy (ESGE) guidelines were updated in 2020 1 2 5 . The UK guideline defines high risk patients as those with: ≥ 2 PMPs, of which ≥ 1 is a serrated polyp (or adenoma) ≥ 10 mm or with (high grade) dysplasia; ≥ 5 PMPs; or ≥ 1 large (≥ 20 mm) nonpedunculated PMP; surveillance colonoscopy at 3 years is recommended for these patients. Low risk patients without these findings are encouraged to participate in routine CRC screening instead of surveillance. The US and ESGE guidelines recommend surveillance at 3 years for patients with 5–10 PMPs or a PMP ≥ 10 mm or with high grade dysplasia, but generally advise no surveillance or surveillance after a longer interval for patients without these findings 2 5 ; an exception is the US recommendation for surveillance at 3 years in patients with tubulovillous/villous adenomas 2 .

No data exist on the effects of surveillance interval length on long-term post-polypectomy CRC outcomes. A randomized controlled trial examining CRC incidence with different surveillance intervals is underway, although results are not expected before 2029 6 . Longer intervals between baseline and first surveillance are associated with increased odds of detecting CRC or adenomas with advanced features at first surveillance in some studies 7 8 , but not others 9 10 11 12 . We aimed to elucidate effects of interval length on advanced neoplasia detection at surveillance to inform whether recommendations for no surveillance and a 3-year surveillance interval are appropriate for low risk and high risk patients, respectively.

## Methods

Our retrospective study used routine data from 17 UK hospitals on patients undergoing colonoscopy with polypectomy from 1984 to 2010 (87 % from 2000 to 2010). We used this cohort in previous studies examining long-term CRC incidence post-polypectomy 7 13 14 .

To be included, hospitals had to have electronic endoscopy and pathology records for colonic examinations for ≥ 6 years before the study start (2006). We searched endoscopy databases for patients with colonic examinations before the end of 2010 and pathology databases for colorectal lesions. We entered endoscopy and pathology data into a database. When the same polyp was described in multiple records, we combined data using rule hierarchies to create summary values for size, histology, and location 7 .

We examined patients’ records to identify the first adenoma diagnosis, defined as “baseline.” The “baseline visit” included all examinations performed at baseline to completely examine the colon and remove detected lesions, sometimes spanning multiple days.

To be included, patients had to have a colonoscopy and ≥ 1 adenoma at baseline. We excluded patients with the following: CRC or colectomy at/before baseline; Lynch syndrome or family history of familial adenomatous polyposis; polyposis, juvenile polyps, or hamartomatous polyps; inflammatory bowel disease or colitis; colorectal carcinoma in situ reported in registry data > 3 years pre-baseline; or examinations with no date recorded. We additionally excluded, from analysis, patients with insufficient information for risk classification by the UK (2020) guideline 1 , and those with an incomplete colonoscopy, colonoscopy of unknown completeness, or poor bowel preparation at baseline, so that our data reflect present-day colonoscopy practice 15 .

We collected data on colonic examinations performed after the baseline visit through 2016, combining them into surveillance visits 7 . We defined the surveillance interval as the interval between the last most complete examination in one visit to the first examination in the next. Surveillance intervals were categorized as < 18 months (reference group) or 2, 3, 4, 5, or 6 years (± 6 months).

For patients undergoing an examination > 6.5 years after their previous visit, we did not include this as surveillance because we thought it more likely that the patient was re-presenting with symptoms. We chose this cutoff based on the longest interval (5 years) recommended in the 2002 UK guideline (most examinations in our dataset occurred during the era of these guidelines 16 ) and extended it by 1.5 years to allow for endoscopy delays. We excluded patients who did not attend surveillance within 6.5 years.

We classified patients’ risk following the UK (2020) guideline 1 . High risk patients were those who had any of the following at baseline: i) ≥ 2 PMPs, of which ≥ 1 was a serrated polyp (or adenoma) ≥ 10 mm or with (high grade) dysplasia; ii) ≥ 5 PMPs; or iii) ≥ 1 large (≥ 20 mm) nonpedunculated PMP. We defined these as “high risk findings (HRFs)” (the guideline uses this term for the first two groups, and considers large nonpedunculated PMPs separately) 1 . Patients with any HRF were classified as high risk even if they had some PMPs with missing size, shape, or dysplasia information. Patients with no HRFs were classified as low risk.

We examined the proportion of high risk patients who were also classified as high risk by the ESGE (2020) guideline, which defines high risk patients as those with any serrated polyp (or adenoma) ≥ 10 mm or with (high grade) dysplasia, or ≥ 5 adenomas 5 .

Primary outcomes were incident advanced adenomas, HRFs, and CRC detected at first and second surveillance, ascertained using pathology data. Additionally, we obtained data on CRC diagnoses from the National Health Service (NHS) Central Register, National Services Scotland, and NHS Digital through 2016/17 (Scotland/England); any CRCs recorded in these databases but not in pathology data were included as outcomes.

Advanced adenomas were adenomas ≥ 10 mm, with tubulovillous/villous histology, or with high grade dysplasia. HRFs were ≥ 2 PMPs, of which ≥ 1 was a serrated polyp (or adenoma) ≥ 10 mm or with (high grade) dysplasia; ≥ 5 PMPs; or ≥ 1 large (≥ 20 mm) nonpedunculated PMP, based on the UK (2020) guideline 1 . We included hyperplastic polyps and sessile serrated lesions as serrated polyps. The guideline also includes serrated adenomas and mixed hyperplastic-adenomatous polyps as serrated polyps; however, considering the age of our data, we thought such lesions in our data more likely represented adenomas and included them as such 7 .

We defined CRCs as colorectal adenocarcinomas, including cancers with unspecified morphology located between the rectum and cecum (assumed adenocarcinomas), but not those located anally (assumed squamous cell carcinomas). In situ cancers were not included.

### Ethics approval

Ethics approval for our original study involving patients included in the present study was granted by the Royal Free Research Ethics Committee (REC). Further ethics approval for protocol extension was granted by the London – Hampstead REC and the Health Research Authority (HRA; REC reference 06/Q0501/45, IRAS ID 55943). Approval for the processing of patient-identifiable information without consent was originally granted by the Patient Information Advisory Group (PIAG) under Section 60 of the Health and Social Care Act 2001 (re-enacted by Section 251 of the NHS Act 2006), and subsequent amendments/annual reviews were approved by the HRA-Confidentiality Advisory Group (reference PIAG 1–05[e]/2006).

### Statistical analysis

We used chi-squared tests to compare baseline characteristics between low risk and high risk patients, and to examine associations between baseline characteristics and interval length to first surveillance.

Within risk groups, we calculated detection rates (with 95 % confidence intervals [CIs]) of advanced adenomas, HRFs, and CRC at surveillance as the proportion of patients with ≥ 1 of the specified outcome detected. We examined detection rates by interval length from baseline, using univariable modified Poisson regression to calculate risk ratios (RRs) and 95 %CIs. We calculated RRs adjusted for baseline characteristics independently associated with increased detection of advanced adenomas, HRFs, or CRC at first surveillance using multivariable modified Poisson regression. Such characteristics were identified from models including all patients, using backward stepwise selection based on Wald tests to retain variables with *P* values of < 0.05, and considering sex, age, PMP number and size, adenoma histology and dysplasia, proximal polyps, year of baseline visit, and family history of cancer/CRC. We included interval length, our main variable of interest, in all models. Tests for trend were conducted for interval length.

For high risk patients, we examined detection rates of advanced adenomas, HRFs, and CRC at second surveillance by interval from first surveillance, stratifying by presence of HRFs at the first surveillance. There were too few outcomes to perform regression analysis.

We did not compare detection rates between the risk groups because this would not serve our aim of examining the effect of interval within each group.

When calculating advanced adenoma and HRF detection rates at surveillance, we excluded PMPs detected in the same/adjacent colonic segment to baseline PMPs ≥ 15 mm seen at least twice within the preceding 3 years (first surveillance: advanced adenomas, n = 77; HRFs, n = 43; second surveillance: advanced adenomas, n = 23; HRFs, n = 9) because these were likely to have been incompletely resected at baseline and under polypectomy site surveillance; their inclusion would confound associations between interval length and neoplasia detection at surveillance 7 . We excluded patients with CRC from these calculations, given their more advanced diagnosis.

When calculating CRC detection rates at surveillance, we excluded CRCs assumed to have arisen from incompletely resected baseline PMPs, namely those detected in the same/adjacent colonic segment to a baseline PMP ≥ 15 mm seen at least twice within the preceding 5 years (first surveillance: n = 19; second surveillance: n = 5). This approach was taken to account for improvements in endoscopic resection over the past decade, so that our data reflect contemporary practice 17 .

In additional analyses, we assessed the robustness of our results to our choice of reference group and interval cutoff, assigning patients with an interval of 3 years as the reference group and using a cutoff of 4.5 years. We performed additional analyses to assess the effect of adjusting for clustering by hospital in all models.

We conducted analyses in Stata/IC V.13.1 18 . Our significance level was 5 %. The study protocol is available online 19 .

## Results

### Patients

**Fig. 1:**
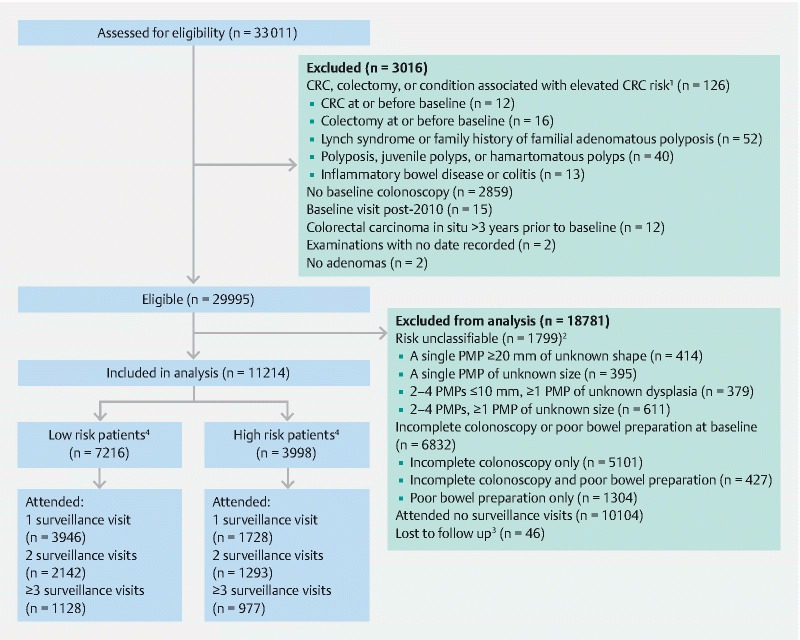
Study profile flow diagram. ^1^ Not mutually exclusive. ^2^ Mutually exclusive groups. Among the 395 patients with a single PMP of unknown size, 90 PMPs were also of unknown shape. Of the 611 patients with 2–4 PMPs and ≥ 1 PMP of unknown size, 99 patients also had ≥ 1 PMP of unknown dysplasia. ^3^ Of the 46 patients lost to follow-up, 22 were lost because they had no surveillance and could not be traced through national data sources, 20 because they had all examinations after emigrating, and 4 because their date of birth was unknown. ^4^ High risk patients were those with any of the following at baseline: ≥ 2 PMPs, of which ≥ 1 was a serrated polyp (or adenoma) ≥ 10 mm or with (high grade) dysplasia; ≥ 5 PMPs; or ≥ 1 large (≥ 20 mm) nonpedunculated PMP. Low risk patients were those with none of these findings at baseline. Of those classified as high risk, 85 % had ≥ 2 PMPs of which ≥ 1 was a serrated polyp (or adenoma) ≥ 10 mm or with (high grade) dysplasia, 8 % had ≥ 5 PMPs only, and 8 % had a large nonpedunculated PMP only. CRC, colorectal cancer; PMP, premalignant polyp.

From 33 011 patients, we excluded 126 with CRC or colectomy at/before baseline, or with a condition associated with elevated CRC risk, 2859 with no baseline colonoscopy, 15 whose baseline visit was after 2010, 12 with colorectal carcinoma in situ > 3 years pre-baseline, 2 with examinations with no date recorded, and 2 without adenomas ( Fig. 1 ). Additionally, we excluded 1799 patients with unclassifiable risk, 6832 whose baseline colonoscopy was not complete or had poor bowel preparation, 10 104 who did not attend surveillance within ≤ 6.5 years after baseline, and 46 who were lost to follow-up. The remaining 11 214 patients who had all attended ≥ 1 surveillance visit within ≤ 6.5 years were included in the analysis ( Fig. 1 ).

A comparison of baseline characteristics among surveillance attenders compared with non-attenders is described elsewhere 15 . Of the 11 214 patients, 7216 (64 %) were classified as low risk and 3998 (36 %) were classified as high risk at baseline ( Fig. 1 ). Baseline characteristics of low risk and high risk patients are shown in Table 1 . Among high risk patients, 3836 (96 %) were also classified as high risk by the ESGE guideline (data not shown) 5 .

**Table 1:** Baseline characteristics by risk group (N = 11 214).

	Low risk patients 1		High risk patients 1	
	n	%	n	%
Total	7216	64.3	3998	35.7
Sex				
Women	3201	44.4	1378	34.5
Men	4015	55.6	2620	65.5
Age in years at baseline				
< 55	1923	26.6	596	14.9
55–64	2337	32.4	1297	32.4
65–74	2171	30.1	1554	38.9
≥ 75	785	10.9	551	13.8
Number of PMPs				
1	5550	76.9	304	7.6
2	1035	14.3	1526	38.2
3	439	6.1	770	19.3
4	192	2.7	406	10.2
≥ 5	0	0.0	992	24.8
PMP size 2 in mm				
< 10	4879	67.6	367	9.2
10–19	1616	22.4	1905	47.6
≥ 20	702	9.7	1704	42.6
Unknown	19	0.3	22	0.6
Adenoma histology 3				
Tubular	4685	64.9	1503	37.6
Tubulovillous	1891	26.2	1926	48.2
Villous	213	3.0	452	11.3
Unknown	427	5.9	117	2.9
Adenoma dysplasia 4				
Low grade	6518	90.3	2952	73.8
High grade	455	6.3	956	23.9
Unknown	243	3.4	90	2.3
Proximal polyps 5				
No	4360	60.4	1532	38.3
Yes	2856	39.6	2466	61.7
Year of baseline visit				
1984–1999	863	12.0	451	11.3
2000–2004	2392	33.1	1174	29.4
2005–2010	3961	54.9	2373	59.4
Family history of cancer/CRC 6				
No	6308	87.4	3783	94.6
Yes	908	12.6	215	5.4

PMP, premalignant polyp; CRC, colorectal cancer.

Comparing baseline characteristics between low risk patients and high risk patients with the chi-squared test, all comparisons had a *P* value < 0.001.

1 High risk patients were those with any of the following at baseline: ≥ 2 PMPs, of which ≥ 1 was an adenoma ≥ 10 mm or with high grade dysplasia, or a serrated polyp ≥ 10 mm or with any dysplasia; ≥ 5 PMPs; or ≥ 1 large (≥ 20 mm) nonpedunculated PMP. Low risk patients were those with none of these findings at baseline.

2 PMP size was defined by the largest PMP reported at baseline.

3 Adenoma histology was defined by the greatest degree of villous architecture reported at baseline.

4 Adenoma dysplasia was defined by the highest grade of dysplasia reported at baseline.

5 Proximal polyps were those proximal to the descending colon.

6 Family history of cancer/CRC was defined as “family history of cancer or CRC reported at an examination before or during visit.” Of those reported to have a “family history of cancer,” 72 % were from a hospital specializing in colorectal diseases and so we assumed that they had a family history of CRC.

### First surveillance

Examining all patients together, the interval from baseline was independently associated with detection rates of advanced adenomas and CRC (multivariable *P* trend < 0.001), but not HRFs (multivariable *P* trend = 0.06), at first surveillance. Baseline characteristics that were independently associated with detection rates were: age, PMP number and size, adenoma histology, proximal polyps, and family history of cancer/CRC for advanced adenomas; sex, PMP number and size, proximal polyps, and year of baseline visit for HRFs; and age and proximal polyps for CRC (see **Table 1 s** in the Supplementary Material).

Among low risk patients, the median time from baseline to first surveillance was 3.0 years (interquartile range [IQR] 2.0–4.1). Baseline characteristics associated with shorter intervals included age ≥ 65 years, 1 or 4 PMPs, PMPs ≥ 10 mm, adenomas with tubulovillous/villous histology or high grade dysplasia, and baseline visits performed pre-2005. Intervals were generally longer in patients with a family history of cancer/CRC than in those without ( **Table 2 s** ).

**Table 2a:** Association between interval length and detection rates of advanced adenomas at first surveillance by risk group.

Interval to first surveillance	n 1	%	Advanced adenomas					
			Cases	% (95 %CI) 2	Univariable RR (95 %CI)	*P* value	Multivariable RR 3 (95 %CI)	*P* value
Low risk patients 4								
Total	7135	100	556	7.8 (7.2–8.4)		0.16 5		0.007 5
< 18 months	1327	18.6	100	7.5 (6.2–9.1)	1	0.03 6	1	< 0.001 6
2 years 7	1120	15.7	71	6.3 (5.0–7.9)	0.84 (0.63–1.13)		0.93 (0.70–1.25)	
3 years 7	2337	32.8	177	7.6 (6.5–8.7)	1.01 (0.79–1.27)		1.12 (0.89–1.42)	
4 years 7	844	11.8	75	8.9 (7.1–11.0)	1.18 (0.89–1.57)		1.40 (1.05–1.86)	
5 years 7	1088	15.2	92	8.5 (6.9–10.3)	1.12 (0.86–1.47)		1.40 (1.06–1.84)	
6 years 7	419	5.9	41	9.8 (7.1–13.0)	1.30 (0.92–1.84)		1.54 (1.09–2.18)	
High risk patients 4								
Total	3940	100	604	15.3 (14.2–16.5)		0.14 5		0.009 5
< 18 months	1528	38.8	229	15.0 (13.2–16.9)	1	0.09 6	1	< 0.001 6
2 years 7	684	17.4	95	13.9 (11.4–16.7)	0.93 (0.74–1.16)		0.97 (0.78–1.21)	
3 years 7	1059	26.9	156	14.7 (12.6–17.0)	0.98 (0.81–1.19)		1.13 (0.94–1.37)	
4 years 7	307	7.8	61	19.9 (15.6–24.8)	1.33 (1.03–1.71)		1.49 (1.16–1.92)	
5 years 7	242	6.1	40	16.5 (12.1–21.8)	1.10 (0.81–1.50)		1.31 (0.96–1.78)	
6 years 7	120	3.0	23	19.2 (12.6–27.4)	1.28 (0.87–1.88)		1.49 (1.01–2.19)	

CI, confidence interval; RR, risk ratio; CRC, colorectal cancer; PMP, premalignant polyp.

1 Only patients without CRC diagnosed at first surveillance were included in the analysis of detection rates of advanced adenomas at first surveillance; patients with CRC were excluded from the analysis given their more advanced diagnosis.

2 Clopper–Pearson exact 95 %CIs.

3 Adjusted for age, number of PMPs, PMP size, adenoma histology, and presence of proximal polyps at baseline and family history of cancer/CRC.

4 High risk patients were those with any of the following at baseline: ≥ 2 PMPs, of which ≥ 1 was an adenoma ≥ 10 mm or with high grade dysplasia, or a serrated polyp ≥ 10 mm or with any dysplasia; ≥ 5 PMPs; or ≥ 1 large (≥ 20 mm) nonpedunculated PMP. Low risk patients were those with none of these findings at baseline.

5 P value calculated with Wald test

6 P value calculated from a test for trend.

7 Interval length ± 6 months.

Among low risk patients, detection rates of advanced adenomas, HRFs, and CRC at first surveillance were 7.8 %, 3.7 %, and 1.1 %, respectively. There was a trend of increasing detection rates of advanced adenomas with increasing interval length (multivariable *P* trend < 0.001), reaching 9.8 % at an interval of 6 years. Detection of HRFs or CRC did not increase with increasing interval length, remaining < 5 % and < 2 %, respectively, with all interval categories (multivariable *P* trend = 0.06 and 0.08, respectively) ( Table 2a , Table 2b , Table 2c ).

**Table 2b:** Association between interval length and detection rates of high risk findings at first surveillance by risk group.

Interval to first surveillance	n 1	%	High risk findings 2					
			Cases	% (95 %CI) 3	Univariable RR (95 %CI)	*P* value	Multivariable RR 4 (95 %CI)	*P* value
Low risk patients 5								
Total	7135	100	261	3.7 (3.2–4.1)		0.58 6		0.51 6
< 18 months	1327	18.6	41	3.1 (2.2–4.2)	1	0.10 7	1	0.06 7
2 years 8	1120	15.7	34	3.0 (2.1–4.2)	0.98 (0.63–1.54)		1.01 (0.65–1.57)	
3 years 8	2337	32.8	91	3.9 (3.1–4.8)	1.26 (0.88–1.81)		1.25 (0.87–1.79)	
4 years 8	844	11.8	35	4.1 (2.9–5.7)	1.34 (0.86–2.09)		1.42 (0.91–2.21)	
5 years 8	1088	15.2	43	4.0 (2.9–5.3)	1.28 (0.84–1.95)		1.32 (0.86–2.03)	
6 years 8	419	5.9	17	4.1 (2.4–6.4)	1.31 (0.75–2.29)		1.40 (0.81–2.44)	
High risk patients 5								
Total	3940	100	393	10.0 (9.1–11.0)		0.13 6		0.16 6
< 18 months	1528	38.8	171	11.2 (9.7–12.9)	1	0.72 7	1	0.31 7
2 years 8	684	17.4	54	7.9 (6.0–10.2)	0.71 (0.53–0.95)		0.75 (0.56–1.00)	
3 years 8	1059	26.9	93	8.8 (7.1–10.7)	0.78 (0.62–1.00)		0.92 (0.72–1.18)	
4 years 8	307	7.8	35	11.4 (8.1–15.5)	1.02 (0.72–1.43)		1.22 (0.87–1.72)	
5 years 8	242	6.1	27	11.2 (7.5–15.8)	1.00 (0.68–1.46)		1.19 (0.81–1.76)	
6 years 8	120	3.0	13	10.8 (5.9–17.8)	0.97 (0.57–1.65)		1.12 (0.66–1.89)	

CI, confidence interval; RR, risk ratio; CRC, colorectal cancer; PMP, premalignant polyp.

1 Only patients without CRC diagnosed at first surveillance were included in the analysis of detection rates of high risk findings at first surveillance; patients with CRC were excluded from the analysis given their more advanced diagnosis.

2 A patient was included as having high risk findings if they had ≥ 2 PMPs, of which ≥ 1 was an adenoma ≥ 10 mm or with high grade dysplasia, or a serrated polyp ≥ 10 mm or with any dysplasia; ≥ 5 PMPs; or ≥ 1 large (≥ 20 mm) nonpedunculated PMP at first surveillance.

3 Clopper–Pearson exact 95%CIs.

4 Adjusted for sex, number of PMPs, PMP size, presence of proximal polyps at baseline and year of baseline visit.

5 High risk patients were those with high risk findings, as defined above, at baseline; low risk patients were those with no high risk findings at baseline.

6 *P* value calculated with Wald test.

7 *P* value calculated from a test for trend.

8 Interval length ± 6 months.

**Table 2c:** Association between interval length and detection rates of colorectal cancer at first surveillance by risk group.

Interval to first surveillance	n	%	Colorectal cancer					
			Cases	% (95 %CI) 1	Univariable RR (95 %CI)	*P* value	Multivariable RR 2 (95 %CI)	*P* value
Low risk patients 3								
Total	7216	100	81	1.1 (0.9–1.4)		0.24 4		0.17 4
< 18 months	1340	18.6	13	1.0 (0.5–1.7)	1	0.34 5	1	0.08 5
2 years 6	1136	15.7	16	1.4 (0.8–2.3)	1.45 (0.70–3.01)		1.52 (0.74–3.13)	
3 years 6	2355	32.6	18	0.8 (0.5–1.2)	0.79 (0.39–1.60)		0.93 (0.46–1.86)	
4 years 6	858	11.9	14	1.6 (0.9–2.7)	1.68 (0.79–3.56)		1.97 (0.93–4.16)	
5 years 6	1101	15.3	13	1.2 (0.6–2.0)	1.22 (0.57–2.61)		1.73 (0.80–3.74)	
6 years 6	426	5.9	7	1.6 (0.7–3.4)	1.69 (0.68–4.22)		1.93 (0.78–4.77)	
High risk patients 3								
Total	3998	100	58	1.5 (1.1–1.9)		0.001 4		< 0.001 4
< 18 months	1540	38.5	12	0.8 (0.4–1.4)	1	< 0.001 5	1	< 0.001 5
2 years 6	697	17.4	13	1.9 (1.0–3.2)	2.39 (1.10–5.22)		2.30 (1.05–5.04)	
3 years 6	1070	26.8	11	1.0 (0.5–1.8)	1.32 (0.58–2.98)		1.54 (0.68–3.48)	
4 years 6	317	7.9	10	3.2 (1.5–5.7)	4.05 (1.76–9.29)		4.44 (1.95–10.08)	
5 years 6	251	6.3	9	3.6 (1.7–6.7)	4.60 (1.96–10.81)		5.80 (2.51–13.40)	
6 years 6	123	3.1	3	2.4 (0.5–7.0)	3.13 (0.90–10.95)		3.96 (1.14–13.71)	

CI, confidence interval; RR, risk ratio; PMP, premalignant polyp.

1 Clopper–Pearson exact 95 %CIs.

2 Adjusted for age and presence of proximal polyps at baseline.

3 High risk patients were those with any of the following at baseline: ≥ 2 PMPs, of which ≥ 1 was an adenoma ≥ 10 mm or with high grade dysplasia, or a serrated polyp ≥ 10 mm or with any dysplasia; ≥ 5 PMPs; or ≥ 1 large (≥ 20 mm) nonpedunculated PMP. Low risk patients were those with none of these findings at baseline.

4 *P* value calculated with Wald test.

5 *P* value calculated from a test for trend.

6 Interval length ± 6 months.

Among high risk patients, the median time from baseline to first surveillance was 2.1 years (IQR 1.1–3.2). Baseline characteristics associated with shorter intervals included age ≥ 65 years, ≥ 5 PMPs, PMPs ≥ 20 mm, adenomas with high grade dysplasia, proximal polyps, and baseline visits performed pre-2000. Intervals were typically longer among those with tubular adenomas at baseline ( **Table 3 s** ).

Among high risk patients, detection rates of advanced adenomas, HRFs, and CRC at first surveillance were 15.3 %, 10.0 %, and 1.5 %, respectively. There was a trend of increasing detection rates of advanced adenomas and CRC with increasing interval length (multivariable *P* trend < 0.001); no such trend was seen for HRFs (multivariable *P* trend = 0.31) ( Table 2a , Table 2b , Table 2c ). For advanced adenomas, the detection rate was similar with intervals of < 18 months (15.0 %), 2 years (13.9 %), and 3 years (14.7 %) but increased to ~20 % with intervals extending to 6 years ( Table 2a ). For CRC, compared with the detection rate with an interval of < 18 months (0.8 %), detection was higher with an interval of 2 years (1.9 %, multivariable RR 2.30, 95 %CI 1.05–5.04), not significantly higher with an interval of 3 years (1.0 %, multivariable RR 1.54, 95 %CI 0.68–3.48), but substantially higher with intervals of 4 years (3.2 %) and 5 years (3.6 %) (multivariable RRs 4.44, 95 %CI 1.95–10.08 and 5.80, 95 %CI 2.51–13.40, respectively) ( Table 2c ). The detection rate did not increase as the interval extended to 6 years, although there were only three CRCs in this category ( Table 2c ).

### Second surveillance

Among high risk patients who, at first surveillance, were free of CRC and had no HRFs detected (n = 3547) ( Table 2b ), 2008 (57 %) attended second surveillance ( Table 3 ). The median time from first to second surveillance in these patients was 3.0 years (IQR 2.0–3.3). At their second surveillance, detection rates of advanced adenomas, HRFs, and CRC were 11.2 %, 8.0 %, and 1.6 %, respectively. Detection rates of advanced adenomas and HRFs did not appear to vary much by interval length from first surveillance. The CRC detection rate tended to increase with increasing interval length, although there were ≤ 8 cases in each interval category ( Table 3 ).

**Table 3:** Detection rates of advanced adenomas, high risk findings, and colorectal cancer at second surveillance among high risk patients, by interval length and presence of high risk findings at first surveillance.

Interval from first to second surveillance			Advanced adenomas 1		High risk findings 1 ^,^ 2		Colorectal cancer	
	n	%	Cases	% (95 %CI) 3	Cases	% (95 %CI) 3	Cases	% (95 %CI) 3
Patients without high risk findings at first surveillance 2								
Total	2008	100	222	11.2 (9.9–12.7)	158	8.0 (6.8–9.3)	32	1.6 (1.1–2.2)
< 18 months	334	16.6	29	8.8 (5.9–12.3)	25	7.6 (4.9–10.9)	3	0.9 (0.2–2.6)
2 years 4	316	15.7	36	11.7 (8.3–15.8)	28	9.1 (6.1–12.9)	8	2.5 (1.1–4.9)
3 years 4	939	46.8	104	11.1 (9.2–13.3)	73	7.8 (6.2–9.7)	6	0.6 (0.2–1.4)
4 years 4	155	7.7	26	17.7 (11.9–24.8)	15	10.2 (5.8–16.3)	8	5.2 (2.3–9.9)
5 years 4	208	10.4	21	10.2 (6.5–15.2)	11	5.4 (2.7–9.4)	3	1.4 (0.3–4.2)
6 years 4	56	2.8	6	11.5 (4.4–23.4)	6	11.5 (4.4–23.4)	4	7.1 (2.0–17.3)
Patients with high risk findings at first surveillance 2								
Total	262	100	51	19.8 (15.1–25.2)	45	17.4 (13.0–22.6)	4	1.5 (0.4–3.9)
< 18 months	108	41.2	16	15.0 (8.8–23.1)	12	11.2 (5.9–18.8)	1	0.9 (0.02–5.1)
2 years 4	57	21.8	13	22.8 (12.7–35.8)	11	19.3 (10.0–31.9)	0	0.0 (–)
3 years 4	68	26.0	13	19.4 (10.8–30.9)	12	17.9 (9.6–29.2)	1	1.5 (0.04–7.9)
4 years 4	14	5.3	4	30.8 (9.1–61.4)	5	38.5 (13.9–68.4)	1	7.1 (0.2–33.9)
5 years 4	10	3.8	2	20.0 (2.5–55.6)	2	20.0 (2.5–55.6)	0	0.0 (–)
6 years 4	5	1.9	3	75.0 (19.4–99.4)	3	75.0 (19.4–99.4)	1	20.0 (0.5–71.6)

CI, confidence interval; CRC, colorectal cancer; PMP, premalignant polyp.

1 Only patients without CRC diagnosed at second surveillance were included in the analyses of detection rates of advanced adenomas and high risk findings at second surveillance; patients with CRC were excluded from the analyses given their more advanced diagnosis.

2 A patient was included as having high risk findings if they had ≥ 2 PMPs, of which ≥ 1 was an adenoma ≥ 10 mm or with high grade dysplasia, or a serrated polyp ≥ 10 mm or with any dysplasia; ≥ 5 PMPs; or ≥ 1 large (≥ 20 mm) nonpedunculated PMP at that surveillance visit.

3 Clopper–Pearson exact 95%CIs were calculated.

4 Interval length ± 6 months.

Among high risk patients who, at first surveillance, were free of CRC but had HRFs detected (n = 393) ( Table 2b ), 262 (67 %) attended second surveillance ( Table 3 ). The median time from first to second surveillance in these patients was 1.9 years (IQR 1.1–3.1). At their second surveillance, detection rates of advanced adenomas, HRFs, and CRC were 19.8 %, 17.4 %, and 1.5 %, respectively. Detection rates of advanced adenomas and HRFs rose above 30 % as the interval extended beyond 3 years, although there were ≤ 5 cases in each interval category. Among these patients, we could not determine an association between interval from first surveillance and CRC detection at second surveillance because only four had CRC at second surveillance ( Table 3 ).

Interpretation of our results did not change when we used patients with a 3-year interval as the reference group, applied an interval cutoff of 4.5 years, or adjusted for clustering by hospital (data not shown).

## Discussion

This is the largest study investigating associations between post-polypectomy surveillance interval length and detection rates of colorectal neoplasia at surveillance, involving > 11 000 patients with ≥ 1 surveillance visit. Classifying patients’ risk following the UK (2020) surveillance guideline 1 , metachronous advanced neoplasia was uncommon among low risk patients, even with surveillance intervals of 6 years, supporting recommendations for no colonoscopy surveillance in these patients. For high risk patients, surveillance at 3 years appears to be optimal for detecting an adequate advanced adenoma yield and ensuring timely CRC detection.

Among low risk patients, CRC detection rates at first surveillance did not vary by interval from baseline, remaining < 2 % even with intervals of 6 years. Advanced adenoma detection rates at first surveillance increased with increasing interval length but remained < 10 %. A yield of 10 % for advanced PMPs has been suggested as a minimum threshold to justify surveillance 1 ; therefore, our results support recommendations for low risk patients to participate in non-invasive CRC screening rather than surveillance 1 20 . This would reduce healthcare costs and unnecessary patient exposure to invasive procedures.

Among high risk patients, the likelihood of detecting CRC at first surveillance increased with increasing interval length. As the interval extended from < 18 months to 2 years, the CRC detection rate increased, but remained < 2 %. A greater increase in CRC detection rate occurred as the interval extended beyond 3 years; with adjustment, the detection rates with an interval of 4 years (3 %) and 5 years (4 %) were four and six times greater, respectively, than with an interval of < 18 months. This indicates that the recommended 3-year interval would help ensure timely CRC detection, preventing progression to advanced stages. Any additional benefit from a shorter interval would be small because CRC detection rates were low with intervals < 3 years. Detection of advanced adenomas among high risk patients increased with increasing interval, although even at 3 years the advanced adenoma yield (15 %) was sufficient to justify surveillance at this interval.

According to the UK (2020) guideline, patients with HRFs at first surveillance should undergo another colonoscopy after 3 years, whereas those with no HRFs can cease surveillance 1 . Most patients entering surveillance are expected to have just one surveillance colonoscopy 1 . Applying these recommendations to our cohort, only 10 % of high risk patients would have been invited for a second surveillance, although our HRF detection rates are likely to be underestimates because serrated polyps were not routinely detected in the era of our data 21 .

In contrast to these expectations 1 , > 50 % of our high risk patients attended ≥ 2 surveillance visits. This is because surveillance regimens in our study were based on physician discretion before publication of the 2002 UK guideline 16 , whereas post-2002, surveillance was recommended until two consecutive negative examinations had been recorded. High risk patients were more likely to attend second surveillance, and to return earlier, if they had HRFs detected at first surveillance. Those with HRFs at first surveillance were more likely to have advanced adenomas or HRFs detected at second surveillance than those without, possibly reflecting different propensities to develop neoplasia or miss/incompletely excise lesions in these two groups.

Although we applied the UK risk criteria 1 , our findings are relevant for surveillance under the ESGE guideline, which classifies risk similarly 5 . The vast majority of “UK high risk” patients were also classified as high risk by the ESGE guideline. It is harder to compare with the US guideline because they classify patients by individual polyp criteria rather than defining low risk and high risk groups 2 .

We had to decide at what point after baseline (or first surveillance) was it more likely that patients were attending examinations because of symptoms rather than for surveillance. As most examinations occurred during the era of the 2002 UK guideline 16 , we applied a cutoff of 6.5 years to the whole cohort, to allow a long enough interval for everyone to return for surveillance, considering delays in endoscopy, but not so long as to capture patients likely to be attending for symptoms.

In our study of post-polypectomy patients classified as “intermediate-risk” by the UK (2002) guideline 7 16 , the odds of detecting incident advanced adenomas and CRC at first surveillance were two- and fourfold greater, respectively, with an interval of 4 years compared with < 18 months, similar to the findings for high risk patients in the present study. Another study reported that among patients with an advanced adenoma or ≥ 3 adenomas at baseline, the odds of detecting advanced adenomas at first surveillance were threefold greater with an interval of ≥ 3 years versus < 3 years, whereas among those with 1–2 adenomas < 10 mm, interval length was not associated with advanced adenoma detection at first surveillance 8 . In other studies, longer intervals were not associated with increased detection of advanced neoplasia at first surveillance 9 10 11 12 . Reasons for interstudy discrepancy might include confounding by inclusion of lesions under polypectomy site surveillance, or if surveillance was performed earlier in patients more likely to have neoplasia found (e. g. those with poor-quality baseline examinations).

No data exist on the effects of surveillance interval on long-term post-polypectomy CRC outcomes. Therefore, while our findings support recommendations for surveillance at 3 years in high risk patients, it remains unknown whether a 3-year interval is superior to longer intervals in terms of long-term protection against CRC. A randomized controlled trial examining long-term CRC incidence with different surveillance intervals will help address this knowledge gap 6 .

Our study has limitations due to its observational and retrospective nature. We were unable to classify the risk of ~1800 patients owing to missing information on baseline polyp characteristics. Classification of serrated polyps was complicated by the evolution in detection and terminology used for these polyps over the study duration. We had incomplete data on reasons for attendance at follow-up examinations and so counted examinations performed within ≤ 6.5 years after the previous visit as surveillance; this might have captured some examinations performed for symptoms. However, our results were robust to changes in our chosen interval cutoff. Some patients might have undergone surveillance at hospitals not included in our study. We had insufficient data to estimate adenoma detection rates for the endoscopists performing the examinations. Associations between interval and neoplasia detection at surveillance might be confounded by baseline characteristics, although we reduced this likelihood by multivariable adjustment. As high risk patients attended first surveillance after a median of 2 years, observed detection rates at first surveillance with intervals of ≥ 3 years are likely to be lower than if intervals more closely aligned with UK (2020) recommendations 1 . Our results should be interpreted with caution because we performed multiple testing and some estimates are imprecise due to few outcomes.

Study strengths include the large size and wide coverage of the UK. We had detailed information on characteristics at baseline colonoscopy, and findings at first and second surveillance, with few missing data. The wide variation in surveillance intervals, owing to the study’s observational and retrospective nature, enabled examination of neoplasia detection rates at many different surveillance intervals; this feature is unique and unlikely to be seen in future studies when adoption of surveillance guidelines is more widespread. Our findings are applicable to contemporary practice because all analyzed patients had a complete baseline colonoscopy.

## Conclusion

Metachronous advanced neoplasia at surveillance was uncommon among low risk patients, even with surveillance intervals extending to 6 years, supporting recommendations for no surveillance in these patients. For high risk patients, whose likelihood of having CRC detected at first surveillance increased with increasing interval length, particularly as the interval extended beyond 3 years, surveillance at 3 years would help to ensure timely detection of CRC.

## Supplementary material

Supplementary material Zusatzmaterial
