# Supplementary material for: Post-polypectomy surveillance interval and advanced neoplasia detection rates: a multicenter, retrospective cohort study
Source: Endoscopy. 2022 Apr 11;54(10):948–58. doi: 10.1055/a-1795-4673 (PMC9500009; doi:10.1055/a-1795-4673)
Supplement: Supplementary file 1 — Supplementary material [file 20538supmat_10-1055-a-1795-4673.pdf]

Supplementary material

Supplementary Material

Post-polypectomy surveillance interval and advanced neoplasia detection rates: a multicenter, retrospective cohort study

Amanda J. Cross, Emma C. Robbins, Kevin Pack, Iain Stenson, Matthew D. Rutter, Andrew M. Veitch, Brian P. Saunders, Stephen W. Duffy, Kate Wooldrage

Supplementary material

Table 1s Detection rates of advanced adenomas, high risk findings, and colorectal cancer at first surveillance, by interval length and baseline characteristics

		Advanced adenomas[a]						High-risk findings[a,b]						Colorectal cancer					
	n	Cases	% (95% CI)[c]	Univariable RR (95% CI)	p-value[d]	Multivariable RR (95% CI)[e]	p-value[d]	Cases	% (95% CI)[c]	Univariable RR (95% CI)	p-value[d]	Multivariable RR (95% CI)[e]	p-value[d]	Cases	% (95% CI)[c]	Univariable RR (95% CI)	p-value[d]	Multivariable RR (95% CI)[e]	p-value[d]
Total	11,214	1,160	10.5 (9.9-11.1)					654	5.9 (5.5-6.4)					139	1.2 (1.0-1.5)				
Interval to first surveillance					0.04		<0.001				0.003		0.09				0.003		<0.001
<18 months	2,880	329	11.5 (10.4-12.8)	1	0.78[f]	1	<0.001[f]	212	7.4 (6.5-8.4)	1	0.02[f]	1	0.06[f]	25	0.9 (0.6-1.3)	1	0.01[f]	1	<0.001[f]
2 years[g]	1,833	166	9.2 (7.9-10.6)	0.80 (0.67-0.95)		0.95 (0.80-1.13)		88	4.9 (3.9-6.0)	0.66 (0.52-0.84)		0.81 (0.64-1.03)		29	1.6 (1.1-2.3)	1.82 (1.07-3.10)		1.94 (1.14-3.29)	
3 years[g]	3,425	333	9.8 (8.8-10.9)	0.85 (0.74-0.98)		1.11 (0.96-1.29)		184	5.4 (4.7-6.2)	0.73 (0.60-0.88)		1.01 (0.83-1.22)		29	0.8 (0.6-1.2)	0.98 (0.57-1.66)		1.22 (0.72-2.07)	
4 years[g]	1,175	136	11.8 (10.0-13.8)	1.03 (0.85-1.24)		1.44 (1.19-1.73)		70	6.1 (4.8-7.6)	0.82 (0.63-1.06)		1.25 (0.96-1.63)		24	2.0 (1.3-3.0)	2.35 (1.35-4.10)		2.94 (1.70-5.11)	
5 years[g]	1,352	132	9.9 (8.4-11.7)	0.86 (0.71-1.04)		1.35 (1.11-1.64)		70	5.3 (4.1-6.6)	0.71 (0.55-0.92)		1.17 (0.89-1.53)		22	1.6 (1.0-2.5)	1.87 (1.06-3.31)		2.83 (1.61-4.98)	
6 years[g]	549	64	11.9 (9.3-14.9)	1.03 (0.80-1.32)		1.50 (1.17-1.93)		30	5.6 (3.8-7.9)	0.75 (0.52-1.09)		1.17 (0.81-1.69)		10	1.8 (0.9-3.3)	2.10 (1.01-4.34)		2.64 (1.28-5.44)	
Sex					0.009		0.28				<0.001		0.03				0.97		0.82
Women	4,579	432	9.6 (8.7-10.4)	1		1		215	4.8 (4.2-5.4)	1		1		57	1.2 (0.9-1.6)	1		1	
Men	6,635	728	11.1 (10.4-11.9)	1.16 (1.04-1.30)		1.06 (0.95-1.19)		439	6.7 (6.1-7.3)	1.41 (1.20-1.65)		1.19 (1.02-1.40)		82	1.2 (1.0-1.5)	0.99 (0.71-1.39)		1.04 (0.74-1.46)	
Age at baseline, years					<0.001		<0.001				0.002		0.27				<0.001		<0.001
<55	2,519	173	6.9 (5.9-8.0)	1		1		111	4.4 (3.7-5.3)	1		1		9	0.4 (0.2-0.7)	1		1	
55-64	3,634	385	10.6 (9.7-11.7)	1.54 (1.30-1.83)		1.31 (1.11-1.56)		214	5.9 (5.2-6.7)	1.34 (1.07-1.67)		1.11 (0.89-1.38)		17	0.5 (0.3-0.7)	1.31 (0.58-2.93)		1.31 (0.59-2.92)	
65-74	3,725	425	11.6 (10.6-12.7)	1.69 (1.42-2.00)		1.37 (1.15-1.63)		238	6.5 (5.7-7.4)	1.47 (1.18-1.83)		1.17 (0.94-1.46)		67	1.8 (1.4-2.3)	5.03 (2.51-10.08)		5.35 (2.67-10.71)	
≥75	1,336	177	13.7 (11.9-15.7)	1.99 (1.63-2.43)		1.58 (1.29-1.92)		91	7.1 (5.7-8.6)	1.60 (1.22-2.09)		1.29 (0.99-1.69)		46	3.4 (2.5-4.6)	9.64 (4.73-19.63)		9.84 (4.82-20.07)	
Number of PMPs					<0.001		<0.001				<0.001		<0.001				0.28		0.67
1	5,854	469	8.1 (7.4-8.8)	1		1		195	3.4 (2.9-3.9)	1		1		60	1.0 (0.8-1.3)	1		1	
2	2,561	263	10.4 (9.3-11.7)	1.29 (1.12-1.49)		1.11 (0.96-1.29)		165	6.5 (5.6-7.6)	1.94 (1.59-2.38)		1.68 (1.37-2.07)		40	1.6 (1.1-2.1)	1.52 (1.02-2.27)		1.36 (0.90-2.04)	
3	1,209	151	12.7 (10.8-14.7)	1.56 (1.32-1.86)		1.25 (1.04-1.50)		86	7.2 (5.8-8.8)	2.14 (1.67-2.74)		1.69 (1.30-2.19)		16	1.3 (0.8-2.1)	1.29 (0.75-2.23)		1.10 (0.63-1.90)	
4	598	99	16.8 (13.9-20.0)	2.07 (1.70-2.53)		1.56 (1.26-1.93)		62	10.5 (8.2-13.3)	3.12 (2.38-4.10)		2.34 (1.75-3.12)		8	1.3 (0.6-2.6)	1.31 (0.63-2.72)		1.07 (0.51-2.26)	
≥5	992	178	18.2 (15.8-20.8)	2.25 (1.92-2.64)		1.66 (1.39-1.99)		146	14.9 (12.8-17.3)	4.44 (3.62-5.44)		3.24 (2.56-4.11)		15	1.5 (0.8-2.5)	1.48 (0.84-2.59)		1.29 (0.70-2.36)	
PMP size, mm[h]					<0.001		<0.001				<0.001		<0.001				0.76		0.92
<10	5,246	395	7.6 (6.9-8.4)	1		1		217	4.2 (3.7-4.8)	1		1		68	1.3 (1.0-1.6)	1		1	
10-19	3,521	413	11.9 (10.8-13.0)	1.55 (1.36-1.77)		1.32 (1.14-1.53)		236	6.8 (6.0-7.7)	1.62 (1.35-1.93)		1.50 (1.25-1.80)		39	1.1 (0.8-1.5)	0.85 (0.58-1.26)		0.96 (0.64-1.43)	
≥20	2,406	344	14.5 (13.1-16.0)	1.90 (1.66-2.18)		1.41 (1.19-1.66)		197	8.3 (7.2-9.5)	1.98 (1.64-2.39)		1.73 (1.42-2.12)		31	1.3 (0.9-1.8)	0.99 (0.65-1.52)		1.02 (0.65-1.62)	
Unknown	41	8	20.0 (9.1-35.6)	2.62 (1.40-4.91)		2.11 (1.12-3.98)		4	10.0 (2.8-23.7)	2.39 (0.93-6.10)		1.45 (0.58-3.64)		1	2.4 (0.1-12.9)	1.88 (0.27-13.23)		1.91 (0.26-14.12)	
Adenoma histology[i]					<0.001		<0.001				<0.001		0.05				0.59		0.76
Tubular	6,188	471	7.7 (7.0-8.4)	1		1		298	4.9 (4.3-5.4)	1		1		73	1.2 (0.9-1.5)	1		1	
Tubulovillous	3,817	520	13.8 (12.7-14.9)	1.79 (1.59-2.01)		1.51 (1.32-1.73)		261	6.9 (6.1-7.8)	1.42 (1.21-1.67)		1.12 (0.94-1.34)		47	1.2 (0.9-1.6)	1.04 (0.73-1.50)		1.04 (0.72-1.50)	
Villous	665	109	16.7 (13.9-19.8)	2.17 (1.79-2.63)		1.70 (1.37-2.10)		61	9.3 (7.2-11.8)	1.92 (1.47-2.49)		1.39 (1.03-1.86)		12	1.8 (0.9-3.1)	1.53 (0.84-2.80)		1.38 (0.75-2.54)	
Unknown	544	60	11.2 (8.6-14.1)	1.45 (1.13-1.87)		1.52 (1.18-1.96)		34	6.3 (4.4-8.7)	1.30 (0.92-1.83)		1.39 (0.99-1.94)		7	1.3 (0.5-2.6)	1.09 (0.50-2.36)		1.17 (0.55-2.53)	

Supplementary material

Adenoma dysplasia[j]					<0.001		0.03				<0.001		0.03				0.05		0.13
Low-grade	9,470	915	9.8 (9.2-10.4)	1		1		517	5.5 (5.1-6.0)	1		1		109	1.2 (0.9-1.4)	1		1	
High-grade	1,411	200	14.5 (12.6-16.4)	1.48 (1.28-1.70)		1.09 (0.94-1.27)		109	7.9 (6.5-9.4)	1.43 (1.17-1.74)		1.08 (0.87-1.33)		27	1.9 (1.3-2.8)	1.66 (1.09-2.52)		1.55 (1.01-2.40)	
Unknown	333	45	13.6 (10.1-17.8)	1.40 (1.06-1.84)		1.43 (1.09-1.89)		28	8.5 (5.7-12.0)	1.54 (1.07-2.21)		1.65 (1.14-2.38)		3	0.9 (0.2-2.6)	0.78 (0.25-2.45)		0.82 (0.26-2.57)	
Proximal polyps[k]					<0.001		<0.001				<0.001		<0.001				0.001		0.007
No	5,892	484	8.3 (7.6-9.0)	1		1		234	4.0 (3.5-4.5)	1		1		54	0.9 (0.7-1.2)	1		1	
Yes	5,322	676	12.9 (12.0-13.8)	1.56 (1.39-1.74)		1.41 (1.25-1.60)		420	8.0 (7.3-8.8)	2.00 (1.71-2.34)		1.49 (1.25-1.77)		85	1.6 (1.3-2.0)	1.74 (1.24-2.45)		1.60 (1.14-2.24)	
Year of baseline visit					0.30		0.32				0.30		0.05				0.91		0.72
1984-1999	1,314	120	9.2 (7.7-10.9)	1		1		70	5.4 (4.2-6.8)	1		1		15	1.1 (0.6-1.9)	1		1	
2000-2004	3,566	376	10.7 (9.7-11.7)	1.16 (0.95-1.41)		1.13 (0.93-1.37)		225	6.4 (5.6-7.3)	1.19 (0.91-1.54)		1.15 (0.89-1.49)		46	1.3 (0.9-1.7)	1.13 (0.63-2.02)		1.05 (0.59-1.89)	
2005-2010	6,334	664	10.6 (9.9-11.4)	1.15 (0.95-1.38)		1.05 (0.87-1.26)		359	5.7 (5.2-6.3)	1.06 (0.83-1.37)		0.94 (0.74-1.21)		78	1.2 (1.0-1.5)	1.08 (0.62-1.87)		0.91 (0.52-1.59)	
Family history of cancer/colo-rectal cancer[l]					<0.001		0.007				0.01		0.24				0.17		0.88
No	10,091	1,093	11.0 (10.4-11.6)	1		1		607	6.1 (5.6-6.6)	1		1		130	1.3 (1.1-1.5)	1		1	
Yes	1,123	67	6.0 (4.7-7.6)	0.55 (0.43-0.70)		0.71 (0.56-0.91)		47	4.2 (3.1-5.6)	0.69 (0.52-0.93)		0.84 (0.63-1.13)		9	0.8 (0.4-1.5)	0.62 (0.32-1.22)		1.05 (0.53-2.12)	

RR=risk ratio. CI=confidence interval. PMP=premalignant polyp.

[a]Only patients without colorectal cancer diagnosed at first surveillance were included in the analyses of detection rates of advanced adenomas and high-risk findings at first surveillance.

[b]A patient was included as having high-risk findings if they had ≥2 premalignant polyps (PMPs), of which ≥1 was an adenoma ≥10mm or with high-grade dysplasia, or a serrated polyp ≥10mm or with any dysplasia; ≥5 PMPs; or ≥1 large (≥20mm) non-pedunculated PMP at first surveillance.

[c]Clopper-Pearson exact 95% confidence intervals.

[d]p-values were calculated with the Wald test.

[e]For the outcome of advanced adenomas, the final multivariable model contained interval length to first surveillance; age, number of PMPs, PMP size, adenoma histology, and presence of proximal polyps at baseline; and family history of cancer/colorectal cancer; adenoma dysplasia was not included in the multivariable model because it was only selected if the unknown category was included. For the outcome of high-risk findings, the final multivariable model contained interval length to first surveillance; sex; number of PMPs, PMP size, and presence of proximal polyps at baseline; and year of baseline visit; interval length was forced into the model; adenoma dysplasia was not included in the multivariable model because it was only selected if the unknown category was included. For the outcome of colorectal cancer, the final multivariable model contained interval length to first surveillance and age and presence of proximal polyps at baseline. For all outcomes, the multivariable RRs for the variables included in the final multivariable model were from the final multivariable model and the p-values were for inclusion of the variable in the model; for the remaining variables, the multivariable RRs were for if the variable was added as an additional variable to the final multivariable model.

[f]p-value calculated from a test for trend.

[g]Interval length ± 6 months.

[h]PMP size was defined by the largest PMP reported at baseline.

[i]Adenoma histology was defined by the greatest degree of villous architecture reported at baseline.

[j]Adenoma dysplasia was defined by the highest grade of dysplasia reported at baseline.

[k]Proximal polyps were those proximal to the descending colon.

[l]Family history of cancer/colorectal cancer was defined as 'family history of cancer or colorectal cancer reported at an examination before or during visit'. Of those reported to have a 'family history of cancer', 72% were from a hospital specialising in colorectal diseases and so we assumed that they had a family history of colorectal cancer.

Supplementary material

Table 2s Interval length to first surveillance in low risk patients, by baseline characteristics

			Interval to first surveillance												
			<18 months		2 years[a]		3 years[a]		4 years[a]		5 years[a]		6 years[a]		
	n	(%)	n	(%)	n	(%)	n	(%)	n	(%)	n	(%)	n	(%)	p-value[b]
Total	7,216		1,340	(18.6)	1,136	(15.7)	2,355	(32.6)	858	(11.9)	1,101	(15.3)	426	(5.9)	
Sex															0.65
Women	3,201	(44.4)	572	(42.7)	521	(45.9)	1,036	(44.0)	387	(45.1)	489	(44.4)	196	(46.0)	
Men	4,015	(55.6)	768	(57.3)	615	(54.1)	1,319	(56.0)	471	(54.9)	612	(55.6)	230	(54.0)	
Age at baseline, years															<0.001
<55	1,923	(26.6)	327	(24.4)	275	(24.2)	593	(25.2)	236	(27.5)	378	(34.3)	114	(26.8)	
55-64	2,337	(32.4)	369	(27.5)	341	(30.0)	790	(33.5)	290	(33.8)	405	(36.8)	142	(33.3)	
65-74	2,171	(30.1)	426	(31.8)	363	(32.0)	788	(33.5)	245	(28.6)	235	(21.3)	114	(26.8)	
≥75	785	(10.9)	218	(16.3)	157	(13.8)	184	(7.8)	87	(10.1)	83	(7.5)	56	(13.1)	
Number of PMPs															<0.001
1	5,550	(76.9)	1,100	(82.1)	876	(77.1)	1,780	(75.6)	654	(76.2)	807	(73.3)	333	(78.2)	
2	1,035	(14.3)	143	(10.7)	174	(15.3)	340	(14.4)	130	(15.2)	184	(16.7)	64	(15.0)	
3	439	(6.1)	53	(4.0)	60	(5.3)	170	(7.2)	51	(5.9)	85	(7.7)	20	(4.7)	
4	192	(2.7)	44	(3.3)	26	(2.3)	65	(2.8)	23	(2.7)	25	(2.3)	9	(2.1)	
PMP size, mm[c]															<0.001
<10	4,879	(67.6)	761	(56.8)	788	(69.4)	1,444	(61.3)	645	(75.2)	911	(82.7)	330	(77.5)	
10-19	1,616	(22.4)	356	(26.6)	240	(21.1)	645	(27.4)	155	(18.1)	150	(13.6)	70	(16.4)	
≥20	702	(9.7)	213	(15.9)	107	(9.4)	263	(11.2)	55	(6.4)	38	(3.5)	26	(6.1)	
Unknown	19	(0.3)	10	(0.7)	1	(0.1)	3	(0.1)	3	(0.3)	2	(0.2)	0	(0.0)	
Adenoma histology[d]															<0.001
Tubular	4,685	(64.9)	741	(55.3)	744	(65.5)	1,489	(63.2)	605	(70.5)	803	(72.9)	303	(71.1)	
Tubulovillous	1,891	(26.2)	427	(31.9)	294	(25.9)	662	(28.1)	195	(22.7)	219	(19.9)	94	(22.1)	
Villous	213	(3.0)	60	(4.5)	35	(3.1)	84	(3.6)	11	(1.3)	16	(1.5)	7	(1.6)	
Unknown	427	(5.9)	112	(8.4)	63	(5.5)	120	(5.1)	47	(5.5)	63	(5.7)	22	(5.2)	
Adenoma dysplasia[e]															<0.001
Low-grade	6,518	(90.3)	1,115	(83.2)	989	(87.1)	2,159	(91.7)	800	(93.2)	1,051	(95.5)	404	(94.8)	
High-grade	455	(6.3)	151	(11.3)	94	(8.3)	143	(6.1)	32	(3.7)	22	(2.0)	13	(3.1)	
Unknown	243	(3.4)	74	(5.5)	53	(4.7)	53	(2.3)	26	(3.0)	28	(2.5)	9	(2.1)	
Proximal polyps[f]															0.49
No	4,360	(60.4)	826	(61.6)	683	(60.1)	1,444	(61.3)	508	(59.2)	641	(58.2)	258	(60.6)	
Yes	2,856	(39.6)	514	(38.4)	453	(39.9)	911	(38.7)	350	(40.8)	460	(41.8)	168	(39.4)	
Year of baseline visit															<0.001
1984-1999	863	(12.0)	307	(22.9)	178	(15.7)	199	(8.5)	98	(11.4)	47	(4.3)	34	(8.0)	
2000-2004	2,392	(33.1)	494	(36.9)	384	(33.8)	784	(33.3)	236	(27.5)	348	(31.6)	146	(34.3)	

Supplementary material

2005-2010	3,961	(54.9)	539	(40.2)	574	(50.5)	1,372	(58.3)	524	(61.1)	706	(64.1)	246	(57.7)	
Family history of cancer/colorectal cancer[g]															<0.001
No	6,308	(87.4)	1,250	(93.3)	1,011	(89.0)	2,003	(85.1)	731	(85.2)	935	(84.9)	378	(88.7)	
Yes	908	(12.6)	90	(6.7)	125	(11.0)	352	(14.9)	127	(14.8)	166	(15.1)	48	(11.3)	

PMP=premalignant polyp.

[a]Interval length ± 6 months.

[b]P-values were calculated with the chi-square test.

[c]PMP size was defined by the largest PMP reported at baseline.

[d]Adenoma histology was defined by the greatest degree of villous architecture reported at baseline.

[e]Adenoma dysplasia was defined by the highest grade of dysplasia reported at baseline.

[f]Proximal polyps were those proximal to the descending colon.

[g]Family history of cancer/colorectal cancer was defined as 'family history of cancer or colorectal cancer reported at an examination before or during visit'. Of those reported to have a 'family history of cancer', 72% were from a hospital specialising in colorectal diseases and so we assumed that they had a family history of colorectal cancer.

Supplementary material

Table 3s Interval length to first surveillance in high risk patients, by baseline characteristics

			Interval to first surveillance												
			<18 months		2 years[a]		3 years[a]		4 years[a]		5 years[a]		6 years[a]		
	n	(%)	n	(%)	n	(%)	n	(%)	n	(%)	n	(%)	n	(%)	p-value[b]
Total	3,998		1,540	(38.5)	697	(17.4)	1,070	(26.8)	317	(7.9)	251	(6.3)	123	(3.1)	
Sex															0.11
Women	1,378	(34.5)	498	(32.3)	247	(35.4)	368	(34.4)	127	(40.1)	94	(37.5)	44	(35.8)	
Men	2,620	(65.5)	1,042	(67.7)	450	(64.6)	702	(65.6)	190	(59.9)	157	(62.5)	79	(64.2)	
Age at baseline, years															<0.001
<55	596	(14.9)	187	(12.1)	110	(15.8)	162	(15.1)	61	(19.2)	52	(20.7)	24	(19.5)	
55-64	1,297	(32.4)	491	(31.9)	195	(28.0)	359	(33.6)	107	(33.8)	93	(37.1)	52	(42.3)	
65-74	1,554	(38.9)	629	(40.8)	264	(37.9)	447	(41.8)	100	(31.5)	80	(31.9)	34	(27.6)	
≥75	551	(13.8)	233	(15.1)	128	(18.4)	102	(9.5)	49	(15.5)	26	(10.4)	13	(10.6)	
Number of PMPs															<0.001
1	304	(7.6)	111	(7.2)	75	(10.8)	70	(6.5)	34	(10.7)	11	(4.4)	3	(2.4)	
2	1,526	(38.2)	469	(30.5)	238	(34.1)	509	(47.6)	136	(42.9)	121	(48.2)	53	(43.1)	
3	770	(19.3)	313	(20.3)	137	(19.7)	186	(17.4)	62	(19.6)	44	(17.5)	28	(22.8)	
4	406	(10.2)	168	(10.9)	71	(10.2)	106	(9.9)	19	(6.0)	26	(10.4)	16	(13.0)	
≥5	992	(24.8)	479	(31.1)	176	(25.3)	199	(18.6)	66	(20.8)	49	(19.5)	23	(18.7)	
PMP size, mm[c]															<0.001
<10	367	(9.2)	141	(9.2)	54	(7.7)	101	(9.4)	24	(7.6)	31	(12.4)	16	(13.0)	
10-19	1,905	(47.6)	668	(43.4)	296	(42.5)	581	(54.3)	161	(50.8)	138	(55.0)	61	(49.6)	
≥20	1,704	(42.6)	720	(46.8)	343	(49.2)	384	(35.9)	130	(41.0)	81	(32.3)	46	(37.4)	
Unknown	22	(0.6)	11	(0.7)	4	(0.6)	4	(0.4)	2	(0.6)	1	(0.4)	0	(0.0)	
Adenoma histology[d]															<0.001
Tubular	1,503	(37.6)	544	(35.3)	249	(35.7)	443	(41.4)	109	(34.4)	103	(41.0)	55	(44.7)	
Tubulovillous	1,926	(48.2)	760	(49.4)	318	(45.6)	515	(48.1)	167	(52.7)	111	(44.2)	55	(44.7)	
Villous	452	(11.3)	183	(11.9)	107	(15.4)	87	(8.1)	37	(11.7)	28	(11.2)	10	(8.1)	
Unknown	117	(2.9)	53	(3.4)	23	(3.3)	25	(2.3)	4	(1.3)	9	(3.6)	3	(2.4)	
Adenoma dysplasia[e]															<0.001
Low-grade	2,952	(73.8)	1,078	(70.0)	488	(70.0)	863	(80.7)	232	(73.2)	199	(79.3)	92	(74.8)	
High-grade	956	(23.9)	423	(27.5)	196	(28.1)	188	(17.6)	74	(23.3)	47	(18.7)	28	(22.8)	
Unknown	90	(2.3)	39	(2.5)	13	(1.9)	19	(1.8)	11	(3.5)	5	(2.0)	3	(2.4)	
Proximal polyps[f]															<0.001
No	1,532	(38.3)	478	(31.0)	274	(39.3)	457	(42.7)	150	(47.3)	119	(47.4)	54	(43.9)	
Yes	2,466	(61.7)	1,062	(69.0)	423	(60.7)	613	(57.3)	167	(52.7)	132	(52.6)	69	(56.1)	
Year of baseline visit															<0.001
1984-1999	451	(11.3)	222	(14.4)	102	(14.6)	66	(6.2)	32	(10.1)	13	(5.2)	16	(13.0)	

Supplementary material

2000-2004	1,174	(29.4)	434	(28.2)	239	(34.3)	304	(28.4)	63	(19.9)	84	(33.5)	50	(40.7)	
2005-2010	2,373	(59.4)	884	(57.4)	356	(51.1)	700	(65.4)	222	(70.0)	154	(61.4)	57	(46.3)	
Family history of cancer/colorectal cancer[g]															0.56
No	3,783	(94.6)	1,466	(95.2)	653	(93.7)	1,007	(94.1)	301	(95.0)	237	(94.4)	119	(96.7)	
Yes	215	(5.4)	74	(4.8)	44	(6.3)	63	(5.9)	16	(5.0)	14	(5.6)	4	(3.3)	

PMP=premalignant polyp.
[a]Interval length ± 6 months.
[b]P-values were calculated with the chi-square test.
[c]PMP size was defined by the largest PMP reported at baseline.
[d]Adenoma histology was defined by the greatest degree of villous architecture reported at baseline.
[e]Adenoma dysplasia was defined by the highest grade of dysplasia reported at baseline.
[f]Proximal polyps were those proximal to the descending colon.
[g]Family history of cancer/colorectal cancer was defined as 'family history of cancer or colorectal cancer reported at an examination before or during visit'. Of those reported to have a 'family history of cancer', 72% were from a hospital specialising in colorectal diseases and so we assumed that they had a family history of colorectal cancer.
